# Safety and Efficacy of Thermal Ablation for Cervical Lymph Node Metastasis from Papillary Thyroid Carcinoma: A Systematic Review of Available Evidence

**DOI:** 10.3390/medsci14050584

**Published:** 2026-09-18

**Authors:** Ilias Karakitsos, Stefanos Triaridis, Petros Karkos

**Affiliations:** 1st Department of Otorhinolaryngology-Head and Neck Surgery, AHEPA University General Hospital, Aristotle University of Thessaloniki, 54636 Thessaloniki, Greece

**Keywords:** radiofrequency ablation, microwave ablation, laser ablation, papillary thyroid carcinoma

## Abstract

Background/Objectives: Cervical lymph node metastasis and nodal recurrence remain clinically significant problems in papillary thyroid carcinoma (PTC). Thermal ablation techniques, including laser ablation (LA), radiofrequency ablation (RFA), and microwave ablation (MWA), have increasingly been investigated as minimally invasive alternatives to repeat surgery, when the latter is rejected by patients or is associated with high morbidity and technical challenges. The aim of this systematic review was to analyze the available evidence on efficacy and safety of LA, RFA, and MWA for cervical lymph node metastasis from PTC. Methods: A literature search was conducted in PubMed, Embase, and Cochrane Library, and extracted outcomes included technical success, volume reduction rate (VRR), complete disappearance rate (CDR), serum thyroglobulin (Tg) response, local recurrence, and complications. Twenty-nine (N = 29) studies were included, eight on LA (*n* = 8), eleven on RFA (*n* = 11), and ten on MWA (*n* = 10). Results: Across the included studies, all three modalities showed high technical success, progressive reduction in nodal volume, and meaningful local disease control. Safety outcomes were generally favorable across all three approaches, with most complications being mild, transient, and resolving in limited time periods. Conclusions: Thermal ablation appears to be an effective and safe treatment option for selected patients with cervical lymph node metastasis from PTC, especially when repeat surgery is undesirable or high risk.

## 1. Introduction

Papillary thyroid carcinoma (PTC) accounts for the vast majority of differentiated thyroid cancers, representing an excellent disease-specific prognosis due to its biological behavior. Nevertheless, persistent or recurrent cervical lymph node metastasis is predominant among PTC patients and remains a clinically significant problem, posing several therapeutic challenges. Although surgery is usually used as a first-line therapeutic option, it is often associated with increased burden and distress due to complications and elevated costs. In this setting, thermal ablation is gaining a growing interest as a safer, efficient, and minimally invasive technique in clinical practice. This alternative to surgical resection has moved from the management of benign thyroid nodules to selected malignant thyroid indications, including cervical lymph node metastases from PTC, with its efficacy and safety being supported both by recent guideline-related sources and available empirical evidence. Although thermal ablation is not yet treated as a full replacement for surgery in all patients, its distinctive therapeutic modalities can provide effective local control with a relatively low complication burden in appropriately selected cases.

## 2. Literature Review

### 2.1. Papillary Thyroid Carcinoma: Epidemiology, Metastasis, and Prognosis

Papillary thyroid carcinoma (PTC) is the most common endocrine cancer and the dominant histological subtype of thyroid cancer, accounting for approximately 80–85% of thyroid malignancies [1]. It commonly spreads through the lymphatic system, making cervical lymph node metastasis a frequent clinical feature [2]. Nodal spread usually begins in the central compartment before progressing to the lateral cervical lymph nodes. Reported rates of central lymph node metastasis vary widely, from 20% to 90%, reflecting differences in tumor characteristics, imaging sensitivity, and detection of micrometastases [3,4]. Although PTC generally has an excellent prognosis, 5–20% of patients may experience recurrence after surgery, often requiring further management [5]. Despite its tendency for nodal spread, PTC is usually associated with very high survival rates, even in the presence of regional lymph node involvement. The main clinical challenge is therefore not survival, but the management of locoregional disease, recurrence, repeat interventions, and long-term surveillance. Recent evidence suggests that recurrence occurs in approximately 3–10% of lower-risk patients, with around 70% of recurrences presenting as nodal metastases, mainly in the cervical compartments [6]. Long-term recurrence risk may also increase over time, with reported recurrence rates of 11.3%, 21.8%, and 29.4% at 10, 20, and 30 years after curative initial surgery, respectively [7]. This long-term burden explains the growing interest in minimally invasive local therapies for recurrent or metastatic cervical lymph node disease in PTC.

### 2.2. Clinical Rationale for Thermal Ablation

Thyroid nodules are discrete and radiologically distinct lesions within the thyroid parenchyma, being characterized by different cytological and sonographic futures, coupled with varied biological behavior [8]. Their majority comprises small intra-thyroidal benign and indolent lesions, which can be managed conservatively with a very favorable prognosis [9]. Although surgery is considered as the first line therapeutic option, there is evidence that less invasive approaches can provide similar results in terms of efficacy, while reducing complications risks and associated follow-up or financial burden [10]. Thyroid thermal ablation, including radiofrequency ablation (RFA), laser ablation (LA), and microwave ablation (MWA), was first introduced during the early 2000s, and since then, it has become an effective and preferable alternative to surgery for treating selected thyroid nodules.

The rationale for thermal ablation lies in various perspectives. First, repeat surgeries for thyroid nodules can be challenging for PTC patients, increasing the risk of serious complications due to postoperative fibrosis and tissue deformation [11]. Indeed, empirical evidence has shown that laryngeal nerve paralysis and hypoparathyroidism are frequent complications in surgical treatment, resulting in impaired quality of life and post-operation dissatisfaction [12]. Second, repeat surgery can cause various distressful symptoms (e.g., high perceived risk of nerve injury) and cosmetic concerns for PTC patients (e.g., operative scarring), as well as financial burden, resulting in surgery decline. Third, there is empirical evidence suggesting that thermal ablation is an efficient and safe alternative to surgical resection, particularly as regards volume reduction rate (VRR), complete disappearance rate (CDR), and associated complications [13]. For example, a recent systematic review evaluating efficacy and safety of RFA and MWA for treating cervical lymph node metastasis from PTC, including 11 articles and involving 233 patients, showed a 95.24% pooled VRR, a 63.1% CDR, and 1.6% recurrence rate [14]. In another review including 33 studies, Chatzisouleiman et al. [15] found a VRR up to 99% at 12-month follow-up in the vast majority of patients, with a CDR exceeding 80% in most studies, and minimal complications, mainly involving temporary regional discomfort, voice change, and hematoma. With this empirical evidence growing, comprehensive research on the efficacy and safety of thermal ablation in PTC patients is still in progress.

### 2.3. Thermal Ablation Therapeutic Modalities

#### 2.3.1. Laser Ablation

LA represents the earliest thermal ablation modality systematically applied to cervical lymph node metastases from PTC, playing a foundational role in establishing thermal ablation as a minimally invasive local treatment for recurrent thyroid cancer. In technical terms, LA involves ultrasound-guided percutaneous insertion of one or more optical fibers into the target lesion, through which laser energy is delivered, inducing localized thermal coagulative necrosis [16]. Thus, LA results in progressive volume reduction and, in many cases, complete lymph node disappearance. Lesions located near critical neck structures (e.g., recurrent laryngeal nerve, trachea, carotid sheath) are highly suitable for LA treatment, which in turn can achieve favorable clinical outcomes safely and without the need for open surgery [17]. With earlier studies focusing on small, well-defined, and anatomically favorable nodal recurrences, subsequent empirical information strengthened the available evidence by documenting that LA can be applied reproducibly in a larger patient cohort [18]. When compared with other thermal ablation modalities, it has been shown that LA may achieve lower volume reduction rates than RFA or MWA, although this difference can be attributed to its selective nature, meaning that LA is usually applied in a smaller ablation field and to strictly selected patients [19].

#### 2.3.2. Radiofrequency Ablation

RFA is now considered as the most established thermal ablation modality for recurrent or persistent PTC, particularly for structurally visible metastatic lymph nodes and thyroid bed recurrences in patients who are poor candidates for reoperation due to possible complications. It has been found that, among the three therapeutic methods, RFA represents the highest proportion of complete disappearance of tumors (76.2%) and the lowest one for recurrence (0.01%) in the case of primary papillary thyroid microcarcinoma [20]. In clinical practice, RFA includes ultrasound-guided percutaneous insertion of an electrode, delivery of alternating high-frequency current, and generation of frictional heat within tissue, producing coagulative necrosis followed by gradual fibrosis and volume shrinkage [21]. In thyroid cancer, although RFA’s efficacy can be limited by carbonization of target tissue and by the heat sink effect [22], recent developments have significantly increased its safety and revolutionized the management of thyroid nodules [23]. Moreover, RFA has become the most “guideline-visible” therapeutic option in recurrent thyroid cancer, accumulating the largest body of recurrence and long-term follow-up data, and providing the strongest technical standardization and clinical guidance [24].

#### 2.3.3. Microwave Ablation

MWA is an ultrasound-guided thermal ablation and minimally invasive therapeutic technique, destroying metastatic cervical lymph nodes from PTC by using electromagnetic energy through a percutaneously inserted antenna. By inserting water molecules within the tissue of the node, rapid coagulative necrosis is generated, resulting in fibrotic shrinkage [25]. In thyroid cancer, local anesthesia is delivered, and subsequently, by real-time ultrasound guidance, the antenna is placed, while following protective maneuvers in order to enhance safety, such as hydrodissection, particularly when lesions are close to the recurrent laryngeal nerve, trachea, esophagus, carotid sheath, or internal jugular vein [26]. Preliminary experimental evidence has shown that MWA is not only absolutely feasible and safe, but also efficient in treating lymph nodes in terms of volume reduction and biochemical improvement, especially in patients for whom surgery is not viable or rejected [27].

Current clinical guidelines recommend thermal ablation modalities as alternative local treatments for carefully selected patients, particularly when repeat surgery is declined, technically challenging, or associated with substantial risk and burden. According to the 2015 American Thyroid Association (ATA) management guidelines, RFA and LA should be considered selectively for biopsy-proven recurrent central lymph nodes ≥ 8 mm and lateral lymph nodes ≥ 10 mm, and for patients at high surgical risk or those refusing further surgery [28]. The 2021 European Thyroid Association and Cardiovascular and Interventional Radiological Society of Europe (ETA/CIRSE) guidelines similarly support minimally invasive treatment for selected radioiodine-refractory cervical recurrences, particularly after previous neck surgery, in patients with small-volume disease and limited lateral lymph node involvement (<4 nodes), after cytological or histological confirmation [29]. Similarly, the 2025 ATA guideline further recognizes RFA as an alternative for recurrent or residual disease, especially when reoperation carries substantial risk, although the recommendation remains conditional and based on low-certainty evidence [8]. The 2025 Korean Society of Thyroid Radiology guideline provides more specific criteria, supporting curative RFA when complete ablation is feasible in patients with ≤3 tumors, each ≤2 cm, with careful assessment and protection of adjacent critical structures [24]. Overall, all available guidelines support that patient selection should be multidisciplinary and based on disease burden, anatomy, previous treatment, radioiodine avidity, tumor behavior, surgical risk, and patient preference.

## 3. Materials and Methods

### 3.1. Literature Search Strategy

The aim of this systematic review is to map and critically discuss the available clinical evidence across the three thermal ablation modalities used in metastatic cervical lymph nodes in PTC. Thus, a structured literature search was designed to identify clinical studies evaluating these procedures in terms of efficacy and safety. This review was performed in accordance with the PRISMA (Preferred Reporting Items for Systematic Reviews and Meta-Analyses) guidelines (see Supplementary Materials). This systematic review was not registered in PROSPERO. A systematic literature search was independently conducted by two reviewers in PubMed/MEDLINE, Embase, and the Cochrane Library from database inception to July 2026. The search terms across the three databases were (“papillary thyroid carcinoma” OR “differentiated thyroid carcinoma” OR PTC) AND (“cervical lymph node metastasis” OR “lymph node metastases” OR “nodal recurrence” OR “locoregional recurrence”) AND (“microwave ablation” OR “radiofrequency ablation” OR “laser ablation” OR “thermal ablation”).

### 3.2. Inclusion and Exclusion Criteria

Studies were included if they met the following criteria: (i) they included patients with PTC with cervical lymph node metastasis, (ii) they evaluated laser ablation, radiofrequency ablation, or microwave ablation as a local treatment for lymph node metastasis, (iii) they reported at least one efficacy or safety outcome, (iv) they were published as full-length peer-reviewed clinical studies, and (v) they included more than ≥5 patients.

Studies were excluded if: (i) they did not involve PTC, (ii) they did not assess thermal ablation focused only on metastatic or recurrent disease in lymph nodes, (iii) they were case reports, (iv) they were reviews, meta-analyses, technical reports, and narrative comments, and (v) they included less than ≤5 patients. All one-to-one comparative studies found were excluded because they concerned primary PTC and no treatment of metastatic cervical lymph nodes.

The methodological quality and potential risk of bias of the included studies were independently assessed by the two reviewers using a relevant checklist including the type of each study (e.g., cohort study, case series, other non-randomized clinical research), participant selection, eligibility criteria, measurement of outcomes, identification of potential confounding factors, completeness of follow-up, and appropriateness of the statistical analyses. Disagreements between the two reviewers were resolved through discussion and consensus, while the quality assessment was used to inform the interpretation of the findings rather than as an additional eligibility criterion.

### 3.3. Data Extraction

The following characteristics were extracted from the included studies: (1) study characteristics: first author, publication year, study period, study design style, thermal ablation method, and sample size; (2) patients characteristics: number, condition, previous therapies; (3) therapeutic efficacy characteristics: technical success, complete ablation, volume reduction rate, complete disappearance rate, changes in lymph node diameter or volume, serum thyroglobulin response, local recurrence, new nodal recurrence, disease progression, and progression-free survival; and (4) safety characteristics: major and minor complications, and toleration.

### 3.4. Included Studies

**Figure 1:**
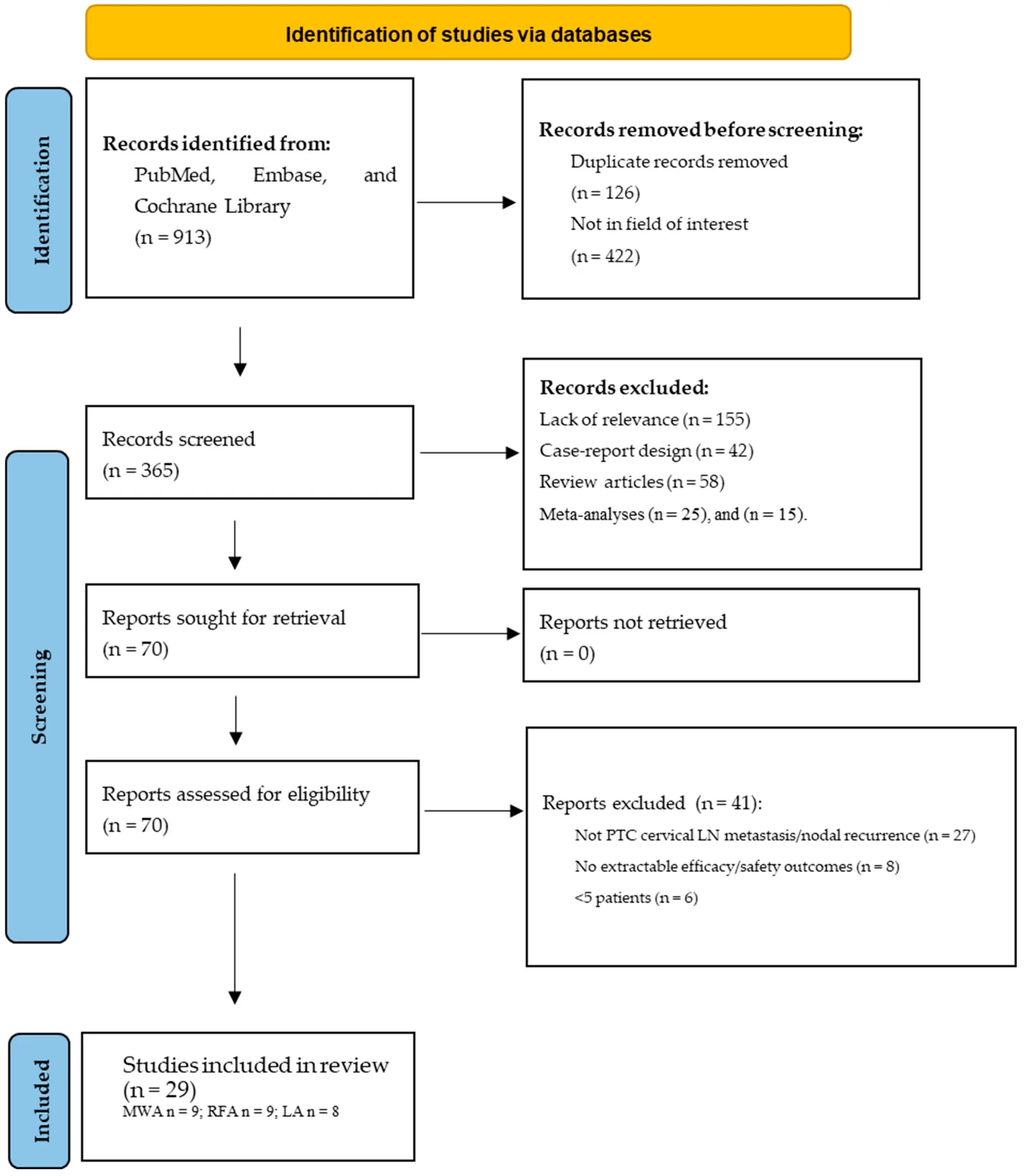
PRISMA 2020 flow diagram of the study selection process.

The initial literature search identified 913 records from PubMed, Embase, and Cochrane Library. After removing 126 duplicate records, and 422 not in the field of interest, 365 studies were screened by title and abstract. Of these, 295 were excluded (unrelated, case reports, reviews, meta-analyses, non-English), and subsequently, 70 full-texts articles were assessed for inclusion. After full-text evaluation, 41 studies were eliminated, 27 because they did not specifically involve papillary thyroid carcinoma with cervical lymph node metastasis or nodal recurrence, 8 because they did not report extractable efficacy or safety outcomes, and 6 because they included less than 5 patients. Finally, 29 studies were reviewed, including 10 studies on microwave ablation, 11 studies on radiofrequency ablation, and 8 studies on laser ablation (see Figure 1). The included studies were predominantly retrospective, single-center observational studies, reflecting the still-developing evidence base for thermal ablation in metastatic or recurrent PTC. Sample sizes were generally small in the earlier literature, but more recent studies, particularly in MWA and RFA, have introduced larger cohorts and longer follow-up periods. The following chart illustrates the literature research process.

## 4. Results

### 4.1. Thermal Ablation Efficacy

#### 4.1.1. Laser Ablation

Across studies, LA is acknowledged as an effective local control procedure for patients who are poor candidates for repeat surgery, providing high technical success, progressive reduction in nodal volume, and meaningful decreases in Tg. In one of the earliest prospective studies, Papini et al. [17] found that the mean nodal volume decreased from 0.64 ± 0.58 mL to 0.07 ± 0.06 mL at 12 months, and reported a VRR at 64.4% and 87.7% at 6 and 12 months, respectively, with Tg becoming undetectable at the last follow-up visit. Mauri et al. [30] showed approximately 93% volume decrease, with complete disappearance in 50% of cases, and Mauri et al. [31] proved that local control was achieved in 40/46 lymph nodes (86.9%), over a mean follow-up of 30 ± 11 months, with no residual disease being seen at imaging in 79.1% of patients. Zhou et al. [32], in 21 patients with 27 recurrent PTC lesions, showed that the largest diameter decreased from 7.5 ± 2.8 mm to 0.4 ± 1 mm, and mean volume decreased from 105.4 ± 114 mm^3^ to 0.8 ± 2.4 mm^3^ at final follow-up.

In 21 metastatic lymph nodes from 17 thyroid cancer patients, Zhang et al. [18] reported that post-laser ablation diameters and volumes decreased significantly compared with baseline, although serum thyroglobulin did not change significantly. Guo et al. [33] evaluated single-fiber LA for selected metastatic lymph nodes from PTC and benign cold thyroid nodules, and in the metastatic PTC lymph node subgroup, found that the lymph node volume decreased from 0.29 ± 0.12 mL to 0.03 ± 0.03 mL at 12 months, with a mean VRR of 90.3 ± 7.6%. In two more recent studies, similar efficacy results are reported. In particular, Offi et al. [34] showed a mean volume reduction of 40.12%, 49.1%, and 59.8% at 1, 3, and 6 months, respectively. Additionally, Yong-Ping et al. [35] displayed a 100% single-ablation success rate, and at the last 6-month follow-up, CDR reached 74.4%, while 11 lesions showed scar-like changes. Furthermore, VRR increased progressively over time, reaching 11.92%, 60.64%, 82.26%, 90.96%, 93.7%, and 97.79% at 6, 12, 18, 24, 30, and 36 months, respectively.

#### 4.1.2. Radiofrequency Ablation

Research evidence shows that RFA can achieve strong local control of selected cervical metastatic or recurrent PTC lesions, as across the studies, RFA generally produced substantial tumor shrinkage, with VRR commonly reported above 90% in longer-followed cohorts, and CDR ranging from approximately 50% to more than 90% depending on lesion size, follow-up duration, and patient group. In the Baek et al. [36] preliminary study, including 10 patients with 12 metastatic tumors, the mean largest diameter decreased from 13.8 ± 7.0 mm to 3.3 ± 3.9 mm, and the mean volume decreased from 55.5 ± 50.3 mm^3^ to 5.7 ± 9.3 mm^3^. Serum thyroglobulin decreased in 7 of 10 patients, suggesting strong biochemical response. In 8 patients with 20 cervical lymph node metastases, 5 treated lymph nodes disappeared, 4 decreased by more than 80%, 9 decreased by 50–80%, and 2 decreased by less than 50% during follow-up [37].

Lim et al. [38] provided stronger evidence for RFA in locoregional recurrent PTC, reporting a mean VRR of 95.1 ± 12.3%, with 82% of recurrent PTC lesions completely disappearing after RFA, along with a significant reduction in serum thyroglobulin, supporting a biochemical response alongside imaging response. In a latter study involving 33 patients with 54 metastatic lymph nodes treated with RFA, Guang et al. [39] verified a time-dependent patter in tumor shrinkage, reporting that the VRR increased from 32.7% at 1 month, to 46.8%, 62.5%, 77.1%, 89.2%, and 94.9% at 3, 6, 12, 18, and 24 months, respectively. Serum thyroglobulin also fell significantly from 10.2 ± 5.1 ng/mL to 1.1 ± 0.8 ng/mL. During a 23.5-month follow-up period and in a sample of 45 PTC patients with 71 metastatic lymph nodes, significant reductions in the VRR were found between every two follow-up visits, while the CDR reached 64.8%, with the remaining 25 metastatic lymph nodes being displayed as small scar-like lesions at the last follow-up visit [40]. Yan et al. [41] provided similar efficacy results in 5 children and adolescents with 10 metastatic lymph nodes, with a mean VRR of 99.28 ± 2.27%, and a CDR of 90.0%.

More recent studies confirm RFA high efficacy rates. In particular, among 24 patients with 37 recurrent PTC lesions followed for at least 10 years, Chung et al. [42] found that the VRR was 99.9%, and the CDR was 91.9%, while recurrence-free survival rates were 86.8%, 75.5%, and 60.6% at 3, 5, and 10 years, respectively. LaForteza et al. [43], in 68 PTC patients with metastatic cervical lymph nodes, reported a median VRR of 79.5%, coupled with a strong biochemical response, as Tg levels significantly declined and 19 patients achieved undetectable thyroglobulin after RFA, although lymph node regrowth occurred in 5 patients over a median follow-up of 18 months. In addition, Thornton et al. [44] examined 45 PTC patients with a total of 71 cervical metastatic lymph nodes, and reported a 64.8% CDR, with 25 lymph nodes (35.2%) remaining as small scar-like lesions. RFA was highly effective in this case, with no lymphatic recurrence at ablated lymph nodes being reported.

Lastly, RFA has been recently compared with standard surgical treatment in terms of efficacy and safety in the treatment of locally recurrent thyroid cancers by two studies. In particular, Choi et al. [45] examined relevant evidence in 96 patients treated with RFA and 125 undergoing repeat surgery, showing similar recurrence-free survival between the two treatment modalities, with the estimated 3-year recurrence-free survival being 100% in both groups, while the 6-year rates were 97.9% and 97.8% following RFA and repeat surgery, respectively. Likewise, changes in serum thyroglobulin did not significantly differ between treatment groups, while overall complications were significantly more frequent following repeat surgery than RFA, with hypocalcemia occurring only in the surgical group. In addition, Xie et al. [46] compared RFA with reoperation for solitary, nerve-adjacent cervical lymph node recurrence of PTC and confirmed that RFA achieved excellent local control, with a 97% VRR at 24 months and complete disappearance of 42.3% of treated lymph nodes, while local recurrence rates were not significantly different from reoperation.

#### 4.1.3. Microwave Ablation

The available evidence suggests that MWA has an almost 100% technical success rate, while across the literature, efficacy is assessed via various metrics, including CDR, VRR, absence of vascular enhancement or local progression, and reduction in serum thyroglobulin. In a preliminary study including 17 patients with 23 recurrent lesions, tumor volume decreased progressively, reaching a mean VRR of 91 ± 14% at 18 months follow-up, while 30.4% of lesions completely disappeared and 52.2% became scar-like [29]. In a mean follow-up of 32 months for a sample of 11 patients with 24 cervical lymph nodes, a complete disappearance rate was detected after MWA, and the mean Tg significantly decreased from an initial 11.81 ± 7.50 ng/mL to 0.43 ± 0.11 ng/mL 3 months after [47]. In 14 patients with recurrent PTC, after the 6-month final follow-up, no incomplete ablation was detected after MWA treatment, and the average largest diameter and volume of the tumors were reduced from 10.1 ± 4.7 mm and 291.9 ± 255.6 mm^3^ to 0.9 ± 1.6 mm and 4.0 ± 9.0 mm^3^, respectively [48].

Cao et al. [49] examined 14 patients with 38 metastatic lymph nodes and reported a significant decrease of serum Tg level at the last post-ablation (median 1.25 ng/mL from initial 8.35 ng/mL), as well as clinically meaningful reductions in metastatic-node size. In the same evidence line, Han et al. [50] showed that the average longest and shortest diameter of the tumors were reduced from 13.21 ± 5.86 mm to 6.74 ± 5.66 mm, and from 9.29 ± 4.09 mm to 4.31 ± 3.56 mm at the final follow-up, with no recurrence at ablated sites being detected. Similar resulted are reported by Xiao et al. [51], who verified a 95.43% VRR for nodes ≤ 10 mm, 87.93% for nodes 10–20 mm, and 81.44% for nodes > 20 mm, and a 63.16%, 80.00%, and 46.67% CDR for the same nodes’ groups, respectively.

In one of the largest and more recent available studies of MWA for cervical lymph node metastasis from PTC, including 131 patients with 535 metastatic lymph nodes, a 100% technical success rate was confirmed, and at the last follow-up, 84.1% of treated nodes had completely disappeared, with no local recurrence or distant metastasis of the ablated target lesions. However, new lymph node recurrence occurred in 22.8% of cases during long-term follow-up [52]. For safety and efficacy enhancement reasons, Wu et al. [53] later improved hydrodissection, reporting no local recurrence and a 75.4% CDR. Lastly, Zhao et al. [54] studied MWA in patients with T1N1M0 PTC and lymph node oligometastasis, finding 1-, 3-, and 5-year progression-free survival rates at 90.2%, 75.3%, and 62.0%, respectively, while Tang et al. [55] retrospectively evaluated 67 patients with cervical metastatic lymph nodes from PTC treated with either MWA (*n* = 19) or repeat surgery (*n* = 48), finding that recurrence-free survival did not differ significantly between the two treatment groups.

### 4.2. Thermal Ablation Safety

#### 4.2.1. Laser Ablation

Available evidence suggests that LA is generally safe and well tolerated, and the most common complications include mild-to-moderate pain, local discomfort, heat sensation, short-lasting hoarseness, and hematoma. In Papini et al. [17], pain was described as tolerable in two cases and mild in three cases, and the only complication was transient dysphonia in one patient. Similarly, Mauri et al. [30,31] reported no major complications, Zhou et al. [32] described the procedure as well tolerated, with 18 of 21 patients being successfully treated in a single session, and Zhang et al. [18] confirmed no significant adverse events. In all of these studies, skin scald, hematoma, pain, and short-lasting voice distortion were among the most frequent minor complications reported, being mostly temporal and resolving within short time periods. Using single-fiber, ultrasound-guided, size-adapted ablation modes, Guo et al. [33] achieved limitation of unnecessary thermal spread in order to eliminate such complications, while Offi et al. [34] also acknowledged no significant complications after performing hydrodissection in a single LA session. Lastly, Yong-Ping et al. [35] reported that 40 out of 43 patients had mild pain, and 3 out of 43 had moderate pain in the ablation region or radiating to the head, ear, shoulder, or teeth, resolving within two weeks without requiring further monitoring. In addition, two patients had transient thyroid-function abnormalities, which normalized without additional treatment.

#### 4.2.2. Radiofrequency Ablation

RFA appears to have a generally favorable safety profile for selected cervical metastatic or recurrent PTC lesions, with the most important potential complication being again voice change/hoarseness, usually related to thermal irritation or injury of the recurrent laryngeal nerve in the central neck. In the preliminary study by Baek et al. [36], no important complications were reported, similar to the results of Wang et al. [37]. Lim et al. [38] also supported RFA as a safe option for locoregional recurrent PTC, and Guang et al. [39] confirmed that RFA is a nonsurgical therapeutic option that can eradicate the lesions with a very low complication rate. The same authors later showed that all 45 patients in their study were successfully treated, without immediate or later major complications occurring [40]. Again, Yan et al. [41] reported that all children and adolescents tolerated RFA well and that no procedure-related complications occurred.

A longer safety profile is provided by Chung et al. [42], who showed no life-threatening or delayed complications during the 10-year follow-up period. LaForteza et al. [43] also reported favorable safety outcomes, reporting only one complication, a transient thermal injury causing Horner’s syndrome, which resolved within 1 month. Lastly, no major complications occurred in the latter U.S. study by Thornton et al. [44], supporting the emerging evidence that RFA can be safely applied to selected nodal recurrences. Overall, all studies suggest that the main safety concern in RFA is transient voice change due to direct thermal injury or hematoma around the nerve. Other complications, such as pain and neck discomfort, skin burn and burning sensation, hematoma, and vagal reaction, are mostly temporal and intraoperative, requiring minimal patient monitoring in longer follow-up periods after the procedure.

#### 4.2.3. Microwave Ablation

MWA studies suggest that this therapeutic modality is usually associated with low major-complication rates, with the most frequent adverse events including mild and temporary local pain, burning sensation, transient dysphonia/hoarseness, vagal reflex, and brief discomfort during ablation. In a sample of 17 patients, only 1 developed transient dysphonia immediately after MWA, and there were no severe or permanent complications [29], although Zhou et al. [48] reported a more cautionary safety signal, suggesting that MWA is less safe for tumors in the central compartment. No complications were reported by Cao et al. [49] and Teng et al. [47]. Safety findings were also favorable in the study by Han et al. [50], with the main intraoperative complaints being burning sensation and pain. Although three patients experienced vagal reflex, no severe safety signal was emphasized, suggesting that most complications in MWA are intraoperative, transient, and manageable.

Minor complications, including mild pain/burning sensation and temporary voice changes, were also reported by Xiao et al. [51], who further showed that complication rates increase with the lymph node size, concluding that larger nodes may be more technically challenging. In a study by Wu et al. [52], the only major complication was transient hoarseness, occurring in 5.3% of ablation sessions/patients, and all cases recovered spontaneously within 6 months. The study also reported one transient episode of heart-rate and blood-pressure decrease, probably due to vagal stimulation, and one case of shoulder pain after ablation of level V nodes, probably related to accessory nerve irritation [50]. In the improved hydrodissection study by Wu et al. [53], hoarseness was again the key major complication, although its occurrence rate was reduced from 8.4% to 4.8% with the improved technique, coupled with a better recovery time (3 versus 6 months). In addition, transient hoarseness occurred in 7.3% of 41 patients in a study by Zhao et al. [54], with all cases recovering without treatment within 2–3 months, and no other complications being encountered. Finally, in a study by Tang et al. [55], total and minor complication rates were higher after repeat surgery compared with the MWA group, whereas major complication rates were not significantly different, while reoperation was also associated with longer hospitalization and higher treatment costs.

The main characteristics and outcomes of the included studies, including study design, number of patients and treated lymph nodes, follow-up duration, efficacy outcomes, and procedure-related complications, are summarized in Table 1 below.

## 5. Discussion

The reviewed evidence suggests that LA, RFA, and MWA all constitute effective, safe, and minimally invasive therapeutic modalities for selected patients with cervical lymph node metastasis from PTC, particularly for those who represent poor candidates for repeat surgery or reject it. However, it should be noted that evidence available for all three approaches differ significantly in maturity, durability, and types of safety or efficacy concerns emphasized. As regards efficacy, LA seems to be a highly effective local-control technique, particularly for small and carefully selected metastatic lymph nodes, with earlier studies showing a marked nodal shrinkage, meaningful local control, and in many cases, complete lesion disappearance [17,30,31,32]. More recent studies confirm this pattern, finding time-dependent increases in VRR and excellent rates of technical success, partly due to delivery of controlled thermal energy, potentially limiting unnecessary thermal spread to adjacent structures [33,34,35]. LA’s tolerability profile is also greatly favorable, with most studies reporting no major complications and transient adverse events including mild pain or hoarseness. However, compared with the other two modalities, LA studies provide less strong evidence, due to the smaller number of treated patients and subsequent limited information on long-term recurrence-free survival rates and durability of biochemical responses.

For RFA, the available evidence is the most mature across all thermal ablation modalities. This approach demonstrates substantial and long-term sustained local control, with VRR values frequently exceeding 90% and CDR ranging from approximately 50% to more than 90%, depending on lesion characteristics, follow-up duration, and patient selection [36,37,38]. More recent studies confirm the time-dependent shrinkage pattern in RFA [39,40], and importantly, 10-year data strengthen the case for RFA as a durable local treatment with excellent long-term volume reduction and high CDR [42]. RFA also has a favorable safety record, with delayed serious complications being extremely uncommon [42] and voice distortion being the most common transient complication in the short-term [38,43]. When performed in selected patients by high-skilled operators, RFA appears highly safe, while providing strong control of ablated lesions, although the risk of new or regional disease progression in the long-term is not eliminated.

Lastly, MWA also demonstrates strong efficacy, and its evidence base appears to be the strongest across the three approaches, because related studies include some of the largest cohorts compared with LA and RFA. Progressive reduction of lesion size and volume, and Tg levels, is a common outcome pattern among studies [29,47,49,50], but recent evidence confirms that VRR and CDR differed across nodal-size categories, with larger nodes having lower CDR and higher complication rates [51]. On the other hand, despite MWA possibly being more technically sensitive in larger or anatomically complex lesions, its safety profile is highly favorable, as this technique is associated with low major-complication rates. In larger and more recent cohorts, transient hoarseness remained the main major complication [52,53,54], signaling the need of adopting careful protective strategies when lesions are large or close to vulnerable structures.

Overall, the available evidence suggests that LA, RFA, and MWA can all achieve meaningful local control in appropriately selected patients with cervical lymph node metastasis or nodal recurrence from PTC. However, comparisons among these modalities should be interpreted cautiously, given that the most of the available studies are retrospective, single-center observational investigations, frequently involving relatively small and highly selected patient populations. Moreover, substantial heterogeneity exists in lesion size and anatomical location, number of treated lymph nodes, previous treatments, follow-up duration, definitions of technical success, criteria for complete disappearance, biochemical endpoints, and methods used to define recurrence, with these differences substantially limiting opportunities for direct comparisons across studies.

Furthermore, surgical reoperation remains the reference treatment, given that it permits comprehensive compartmental clearance of clinically relevant nodal disease and provides histopathological confirmation, while available comparative evidence with thermal ablation remains extremely limited. In the three studies directly comparing thermal ablation with repeat surgical resection, although being retrospective and non-randomized, similar VRR and recurrence-free survival rates were found, suggesting that especially RFA and MWA have comparable efficacy with reoperation [45,46,55]. On the other hand, patients undergoing thermal ablation generally have a smaller number of more localized and technically accessible lesions than patients selected for surgical reoperation, and thus, the available evidence does not permit definitive conclusions regarding therapeutic equivalence between thermal ablation and surgery.

It should also be noted that histological or cytopathological evidence confirming ablation-induced necrosis was limited among the included studies, with ablated metastatic lymph nodes being generally monitored by imaging, thus, adding a limitation to this research. What is more, studies defined technical success or complete ablation according to the absence of enhancement on contrast-enhanced ultrasound or cross-sectional imaging, with complete disappearance and scar-like transformation being treated primarily imaging-based outcomes. A noted exception is the study by Guang et al. [39], in which 54 biopsy-confirmed metastatic cervical lymph nodes from 33 patients were treated with RFA, and at 3 months after ablation, ultrasound-guided core-needle biopsy was systematically performed at both the center and the margin of the ablation zone to assess treatment completeness, confirming no local recurrence at the treated sites. Overall, the effect of thermal ablation on subsequent histopathological assessment constitutes a significant consideration, given that treatment-related changes can persist in lesions that subsequently undergo biopsy or surgical excision and may complicate morphological interpretation. Indeed, histological evaluations of surgically excised thyroid nodules previously treated with thermal ablation have highlighted a wide spectrum of post-ablation alterations, including necrosis, sclerosis, hemorrhagic and ischemic changes, oncocytic features, and nuclear abnormalities [56]. Therefore, thermal ablation should be clearly documented and subsequently histologically assessed, particularly in cases of incomplete response, regrowth, or suspected recurrence.

## 6. Conclusions

Thermal ablation is an effective and generally safe treatment option for selected patients with cervical lymph node metastasis or nodal recurrence from PTC. LA, RFA, and MWA can achieve meaningful nodal shrinkage and local disease control, although the strength of evidence differs across modalities. LA studies are generally based on smaller samples, RFA provides the strongest long-term durability data, and MWA shows promising outcomes, particularly in technical success and complete-disappearance rates. RFA and MWA also appear favorable in terms of Tg reduction, although Tg response should be interpreted cautiously because it may be influenced by factors beyond ablation itself. Overall, LA, RFA, and MWA appear to be acceptable minimally invasive options when performed with appropriate technique and careful patient selection, especially for small, well-visualized metastatic lymph nodes located away from critical structures. However, current evidence does not support the conclusion that one modality is universally superior, as existing studies differ in patient selection, follow-up duration, outcome definitions, design, and sample size. Therefore, the choice between LA, RFA, and MWA should be individualized according to treatment history, nodal size, anatomical location, and available clinical protocols.

## 7. Future Directions

Thermal ablation techniques show high technical success, progressive nodal volume reduction, meaningful local control, and mostly mild, temporary complications. However, future research should move beyond small, single-center and single-arm studies. Prospective multicenter trials are needed to directly compare LA, RFA, and MWA under similar clinical conditions, assessing efficacy, safety, procedure duration, number of sessions, retreatment rates, and need for hydrodissection. Greater standardization of outcome measures, including VRR, CDR, technical efficiency, biological response, recurrence, and complications, is also necessary to allow stronger comparisons across studies. Longer follow-up is equally important, particularly 5- and 10-year assessments, to clarify the durability of local control and the role of thermal ablation in long-term PTC management. Although current evidence suggests good control of treated nodes, recurrence or distant nodal metastasis may still occur. Therefore, thermal ablation should be considered not only as an alternative to surgery, but also as part of a broader multidisciplinary strategy involving active surveillance and individualized treatment planning. Future studies should also define clearer patient and lesion selection criteria by considering nodal size, number of lesions, prior surgeries, previous treatments, and biological response. Further evaluation of technical improvements is needed to strengthen safety profiles, while cost-effectiveness studies should compare LA, RFA, MWA, and repeat surgery in terms of hospitalization, equipment, follow-up, and retreatment needs.

## Figures and Tables

**Table 1:** Characteristics, efficacy, and safety outcomes of studies evaluating thermal ablation for cervical lymph node metastases from papillary thyroid carcinoma.

Study	Modality	Study Design	Patients/Treated LNs	Follow-Up	Main Efficacy Outcomes	Main Complications
Papini et al. [17]	LA	Prospective pilot clinical study	5/8	12 months	Nodal volume 0.64 ± 0.58 → 0.07 ± 0.06 mL; VRR 64.4% at 6 months and 87.7% at 12 months; Tg undetectable at final follow-up	Transient dysphonia in 1 patient; mild/tolerable pain
Mauri et al. [30]	LA	Retrospective analysis of prospectively collected data	15/24	6 and 12 months	≈93% volume reduction; complete disappearance in 50%	No major complications
Mauri et al. [31]	LA	Retrospective single-center study	24/46	30 ± 11 months	Local control in 40/46 LNs (86.9%); no residual disease on imaging in 79.1% of patients	No major complications
Zhou et al. [32]	LA	Retrospective single-center study	21/27	14.9 ± 5.9 months	Largest diameter 7.5 ± 2.8 → 0.4 ± 1.0 mm; volume 105.4 ± 114 → 0.8 ± 2.4 mm^3^	Generally well tolerated; 18/21 treated in one session
Zhang et al. [18]	LA	Prospective observational, single-center study	17/21	17.86 ± 4.70 months	Significant reduction in nodal diameter and volume; no significant Tg change	No significant adverse events
Guo et al. [33]	LA	Prospective single-center preliminary study	8/18	12 months	Volume 0.29 ± 0.12 → 0.03 ± 0.03 mL; VRR 90.3 ± 7.6%	No important thermal-spread complications reported
Offi et al. [34]	LA	Retrospective single-center case series	10/10	6 months	Mean volume reduction 40.12%, 49.1%, and 59.8% at 1, 3, and 6 months	No significant complications; hydrodissection used
Yong-Ping et al. [35]	LA	Retrospective, single-center	43/43	Up to 36 months	100% single-ablation success; CDR 74.4%; VRR increased to 97.79% at 36 months	Mild pain 40/43; moderate pain 3/43; transient thyroid-function abnormalities in 2
Baek et al. [36]	RFA	Retrospective single-center study	10/12	23.0 ± 5.5 months	Diameter 13.8 ± 7.0 → 3.3 ± 3.9 mm; volume 55.5 ± 50.3 → 5.7 ± 9.3 mm^3^; Tg decreased in 7/10	No important complications
Wang et al. [37]	RFA	Prospective clinical study	8/20	9.4 ± 5.1 months	5 LNs disappeared; 4 decreased >80%; 9 decreased 50–80%; 2 decreased <50%	No important complications
Lim et al. [38]	RFA	Retrospective single-center study	39/61	26.4 ± 13.7 months	Mean VRR 95.1 ± 12.3%; CDR 82%; significant Tg reduction	Favorable safety profile
Guang et al. [39]	RFA	Retrospective single-center study	33/54	21 ± 4 months	VRR 32.7% at 1 month → 94.9% at 24 months; Tg 10.2 ± 5.1 → 1.1 ± 0.8 ng/mL	Very-low complication rate
Guang et al. [40]	RFA	Retrospective single-center study	45/71	23.5 months	CDR 64.8%; remaining 25 LNs became small scar-like lesions	No immediate or delayed major complications
Yan et al. [41]	RFA	Retrospective, single-center preliminary study	5/10	52.0 ± 21.4 months	VRR 99.28 ± 2.27%; CDR 90.0%	No procedure-related complications
Chung et al. [42]	RFA	Retrospective single-center long-term cohort study	24/37	≥10 years	VRR 99.9%; CDR 91.9%	No life-threatening or delayed complications
LaForteza et al. [43]	RFA	Retrospective, single-center cohort study	68	Median 18 months	Median VRR 79.5%; significant Tg decline; undetectable Tg in 19 patients	One transient Horner syndrome, resolved within 1 month
Thornton et al. [44]	RFA	Prospective, single-center study	9/10	10.1 months	CDR 64.8%; 25 LNs remained scar-like	No major complications
Yue et al. [27]	MWA	Prospective, single-center study	17/23	18 months	Mean VRR 91 ± 14%; CDR 30.4%; 52.2% became scar-like	Transient dysphonia in 1 patient; no severe/permanent complications
Teng et al. [47]	MWA	Retrospective, single-center study	11/24	Mean 32 months	Complete disappearance reported; Tg 11.81 ± 7.50 → 0.43 ± 0.11 ng/mL at 3 months	No complications reported
Zhou et al. [48]	MWA	Retrospective, single-center preliminary study	14/21	8.4 ± 4.1 months	No incomplete ablation; diameter 10.1 ± 4.7 → 0.9 ± 1.6 mm; volume 291.9 ± 255.6 → 4.0 ± 9.0 mm^3^	Greater safety concern for central-compartment tumors
Cao et al. [49]	MWA	Retrospective, single-center study	14/38	23.6 ± 9.3 months	Tg median 8.35 → 1.25 ng/mL; significant reduction in metastatic-node size	No complications reported
Han et al. [50]	MWA	Retrospective, single-center study	37/98	11.09 ± 9.21 months	Long diameter 13.21 ± 5.86 → 6.74 ± 5.66 mm; short diameter 9.29 ± 4.09 → 4.31 ± 3.56 mm	Burning sensation/pain; vagal reflex in 3 patients; no severe events
Xiao et al. [51]	MWA	Retrospective, single-center study	32/58	12 months	VRR 95.43%, 87.93%, 81.44% for LNs ≤ 10, 10–20, >20 mm; CDR 63.16%, 80.00%, 46.67%	Mild pain/burning, temporary voice changes; complications increased with LN size
Wu et al. [52]	MWA	Retrospective, single-center study	131/535	45.02 ± 18.30 months	Technical success 100%; CDR 84.1%	Transient hoarseness 5.3%; isolated vagal episode and shoulder pain
Wu et al. [53]	MWA	Retrospective, single-center comparative cohort study	266/735	Over 3 months	CDR 75.4%; improved hydrodissection enhanced safety	Hoarseness reduced from 8.4% to 4.8%; recovery reduced from 6 to 3 months
Zhao et al. [54]	MWA	Retrospective, single-center preliminary study	41/61	Median > 3 years	1-, 3-, and 5-year PFS 90.2%, 75.3%, and 62.0%	Transient hoarseness in 7.3%; all recovered in 2–3 months
Choi et al. [45]	RFA vs. repeat surgery	Retrospective, propensity-score matched	96 vs. 125	RFA: 76.8 ± 23.7 months; surgery: 69.1 ± 20.4 months	No significant difference in recurrence	7/70 vs. 27/70; *p* < 0.001; hypocalcemia only after surgery
Teng et al. [47]	MWA vs. repeat surgery	Retrospective, propensity-score matched	17 vs. 17	24.00 ± 12.19 vs. 25.76 ± 14.51 months	No significant difference in recurrence	Total/minor complications higher after surgery; major complications NS
Xie et al. [46]	RFA vs. reoperation	Retrospective, propensity-score matched	71 vs. 71	24 months	No significant difference in recurrence	Overall complications lower with RFA; *p* < 0.001

## Data Availability

No new data were created or analyzed in this study. Data sharing is not applicable to this article.

## Supplementary Materials

The following supporting information can be downloaded at: https://www.mdpi.com/article/10.3390/medsci14050584/s1, PRISMA 2020 checklist.

## Abbreviations

The following abbreviations are used in this manuscript:

LA	Laser Ablation
MWA	Microwave Ablation
RFA	Radiofrequency Ablation
PTC	Papillary Thyroid Carcinoma
CDR	Complete Disappearance Rate
Tg	Thyroglobulin
VRR	Volume Reduction Rate
